# ASSESSING GEROTHERAPEUTIC POTENTIAL OF GLP-1 RECEPTOR AGONISTS

**DOI:** 10.1093/geroni/igae098.0452

**Published:** 2024-12-31

**Authors:** John Newman

**Affiliations:** Buck Institute for Research on Aging, Novato, California, United States

## Abstract

Gerotherapeutics are drugs that target biological mechanisms of aging to achieve broad impacts on multiple chronic diseases of aging as well as geriatric syndromes. No drugs have yet been shown in clinical trials to act as gerotherapeutics. However, several potential candidates have been identified from existing drugs based on proposed interactions with biological mechanisms of aging and supportive data from clinical trials affecting multiple age-related diseases. Metabolism and aging have been mechanistically intertwined since the first rodent studies of caloric restriction a century ago and the identification of the insulin/IGF receptor as the first aging-regulating gene thirty years ago, so it is unsurprising that several candidate gerotherapeutics were first developed for type 2 diabetes (T2DM). Here we will focus on the class of GLP-1 receptor agonists (GLP-1RA), describing preclinical data on interactions with hallmarks of aging that could support a gerotherapeutic role. We will summarize current clinical trial data that includes reduced cardiovascular disease, kidney disease, and mortality in patients with T2DM as well as early or ongoing studies in Alzheimer’s and Parkinson’s disease. We will discuss the limits of current data, priorities for further research, and the known risks of GLP-1RA in considering their development as potential gerotherapeutics.

